# Risk factors associated with delirium in older emergency department patients: A systematic review and meta-analysis

**DOI:** 10.1097/MD.0000000000050157

**Published:** 2026-08-07

**Authors:** Juan Mu, Liqun Zou

**Affiliations:** a Department of Emergency Medicine and West China School of Nursing, West China Hospital, Sichuan University, Chengdu, Sichuan, China.

**Keywords:** delirium, elderly, emergency department, meta-analysis, risk factors

## Abstract

**Background:**

Delirium is a frequent yet underrecognized neuropsychiatric syndrome among older patients presenting to the emergency department (ED) and is associated with adverse outcomes, including prolonged hospitalization, functional decline, and mortality. Evidence on ED delirium risk factors remains fragmented.

**Methods:**

We systematically searched PubMed, Embase, Web of Science, and the Cochrane Library from database inception to December 31, 2025. Comparative observational studies of ED patients aged 65 years or older were eligible if delirium was assessed using a validated tool or diagnostic criteria and if a delirium versus non-delirium comparison was available. Studies enrolling only patients with delirium or lacking a non-delirium comparator were excluded from the risk-factor meta-analysis. Adjusted odds ratios (ORs) were preferentially extracted; unadjusted ORs or raw data were used only when adjusted estimates were unavailable. Random-effects models were used for clinically comparable risk-factor domains.

**Results:**

Sixteen comparative observational studies involving 31,543 older ED patients were included. The pooled delirium prevalence was approximately 10%. Factors significantly associated with delirium included advanced age (OR = 1.49, 95% confidence initerval [CI]: 1.13–1.97), baseline cognitive impairment or dementia (OR = 1.90, 95% CI: 1.31–2.76), and medication-related exposures (OR = 1.49, 95% CI: 1.20–1.85). Functional dependence and ED-related environmental or process factors were not statistically significant in pooled analyses, and comorbidity burden showed a borderline association. Most included studies were of moderate-to-high methodological quality. Heterogeneity was substantial in several domains, and no strong evidence of publication bias was detected for the cognitive impairment analysis.

**Conclusion:**

In older ED patients, delirium is associated with several demographic and clinical factors, particularly age, baseline cognitive impairment, and medication-related exposures. These associations should be interpreted as risk markers rather than causal effects. Early identification of high-risk patients may support risk stratification and guide future delirium prevention research in the ED.

## 1. Introduction

Delirium is a severe and acute neuropsychiatric syndrome characterized by disturbances in attention, awareness, and cognition, with symptoms that fluctuate over time.^[1]^ It is highly prevalent among older adults and is associated with adverse clinical outcomes, including prolonged hospitalization, increased healthcare costs, functional decline, institutionalization, and mortality.^[2,3]^ Older adults frequently present to the emergency department (ED) with confusion, agitation, or behavioral changes, and these symptoms are more often due to underlying medical conditions than to primary psychiatric disorders.^[4]^ As the global population ages, the number of elderly patients presenting to the ED is expected to increase substantially.^[5]^

Delirium is particularly underrecognized in the ED setting, despite reported prevalence rates ranging from 7 to 20%.^[6]^ Studies have shown that delirium is an independent predictor of mortality and long-term cognitive decline.^[7]^ The ED environment itself (including sensory overload, sleep disruption, time pressure, and frequent patient transfers) may further predispose older adults to delirium. Moreover, older patients often have multiple comorbidities, polypharmacy, and atypical clinical presentations, which complicate early detection and management.^[8]^

Early recognition of delirium is essential because it is often reversible when underlying causes are promptly identified and treated. Screening tools such as the confusion assessment method (CAM) for the intensive care unit (ICU) (CAM-ICU) have demonstrated high sensitivity and specificity in the ED setting and significantly increase diagnostic rates compared with routine clinical assessment.^[9,10]^ However, standardized screening remains under-implemented in routine emergency practice.

This systematic review and meta-analysis synthesizes evidence on delirium risk factors in elderly ED patients (≥ 60 years), including both intrinsic and ED-specific contributors, to inform standardized screening, early risk stratification, and tailored interventions, ultimately reducing delirium incidence and improving clinical outcomes.

## 2. Methods

This systematic review and meta-analysis was conducted in accordance with the preferred reporting items for systematic reviews and meta-analyses 2020 statement. Because the study used published aggregate data, no individual patient consent was required. Ethics approval information should be reported according to the target journal’s requirements.

### 2.1. Literature search

A comprehensive electronic search was performed across 4 international databases, including PubMed, Embase, Web of Science, and the Cochrane Library, from database inception to Dec 2025. The search strategy combined subject headings and free-text terms to ensure comprehensiveness, with the core search terms as follows: (“delirium” OR “acute confusional state” OR “acute brain dysfunction”) AND (“emergency department” OR “emergency room” OR “ED”) AND (“elderly” OR “older adults” OR “geriatric patients”). Additionally, the reference lists of all included eligible studies and relevant systematic reviews were manually screened to identify any potentially missed literature. Only studies published in the English language were included in the final analysis.

### 2.2. Study selection and eligibility criteria

#### 2.2.1. Inclusion criteria

Studies were included if they met all of the following prespecified criteria: the study population consisted of ED patients aged 65 years or older, or data for this age group were extractable; the study used a prospective cohort, retrospective cohort, case-control, or cross-sectional comparative design; delirium was diagnosed or screened using validated criteria or tools, such as diagnostic and statistical manual of mental disorders criteria, CAM, brief CAM, CAM-ICU, delirium triage screen, the 4 ‘A’s test, or the delirium observation screening scale; the study compared patients with delirium against patients without delirium; and adjusted odds ratios (ORs) with 95% confidence interval (CIs), unadjusted ORs, or sufficient original data were available for at least 1 candidate risk factor.

#### 2.2.2. Exclusion criteria

Studies were excluded if they met any of the following criteria: narrative reviews, systematic reviews, meta-analyses, editorials, case reports, conference abstracts, or gray literature; pediatric populations, non-ED populations, or studies restricted to ICU, surgical, stroke-unit, or inpatient-ward settings; studies enrolling only patients already diagnosed with delirium, without a non-delirium comparator; studies examining outcomes after delirium diagnosis without baseline risk-factor comparisons; studies without a validated or standardized delirium assessment method; studies with incomplete or unavailable data for effect-size extraction; or overlapping cohorts, in which case the most complete or most recent report was retained.

Two reviewers independently screened titles, abstracts, and full texts. Duplicate records were removed before screening. Disagreements were resolved through discussion or adjudication by a third reviewer.

### 2.3. Data extraction

Two reviewers independently extracted data using a standardized form. Extracted items included the first author, publication year, country, study design, sample size, age threshold, age distribution, sex distribution, delirium assessment method, delirium prevalence or incidence, risk-factor definitions, comparator group, effect estimates, adjustment variables, and data required to calculate ORs and 95% CIs.

For each risk factor, the most fully adjusted OR was preferentially extracted. If multiple adjusted models were reported, the model with the broadest clinically appropriate confounder adjustment was selected, avoiding models that adjusted for variables likely to be mediators. When adjusted estimates were unavailable, unadjusted ORs or raw 2-by-2 data were extracted and labeled as unadjusted.

Risk factors were classified into clinically distinct domains before pooling: demographic factors, baseline cognitive impairment, neurologic or cerebrovascular history, functional dependence/frailty/sensory impairment, comorbidity burden, medication-related exposures, infection or acute illness markers, and ED environmental/process factors. Clinically heterogeneous exposures, such as cerebrovascular disease, cardiovascular disease, malignancy, and multimorbidity, were not pooled as a single composite exposure.^[11–26]^

### 2.4. Quality assessment

The Newcastle–Ottawa scale was used to assess methodological quality across the domains of selection, comparability, and outcome or exposure ascertainment. Studies scoring 7 to 9 points were considered high quality, 4 to 6 points moderate quality, and 0 to 3 points low quality. Quality assessments were performed independently by 2 reviewers, with disagreements resolved by consensus.

### 2.5. Statistical analysis

All statistical analyses were performed using Review Manager 5.4 (The Cochrane Collaboration) and R software, version 4.4.2 (R Foundation for Statistical Computing). ORs and 95% CIs were used as the common effect measure. Adjusted ORs were used preferentially in the primary analyses. Unadjusted estimates were included only when adjusted estimates were not available and were interpreted cautiously. Meta-analysis was performed only for risk-factor domains with at least 3 clinically comparable effect estimates. A random-effects model was used to account for between-study heterogeneity. Heterogeneity was assessed using Cochran *Q* test and the *I*^2^ statistic. Publication bias was evaluated using funnel plots and Egger regression test when the number of included estimates was sufficient. Statistical significance was set at alpha = 0.05 using 2-sided tests.

## 3. Results

### 3.1. Study selection

**Figure 1.**
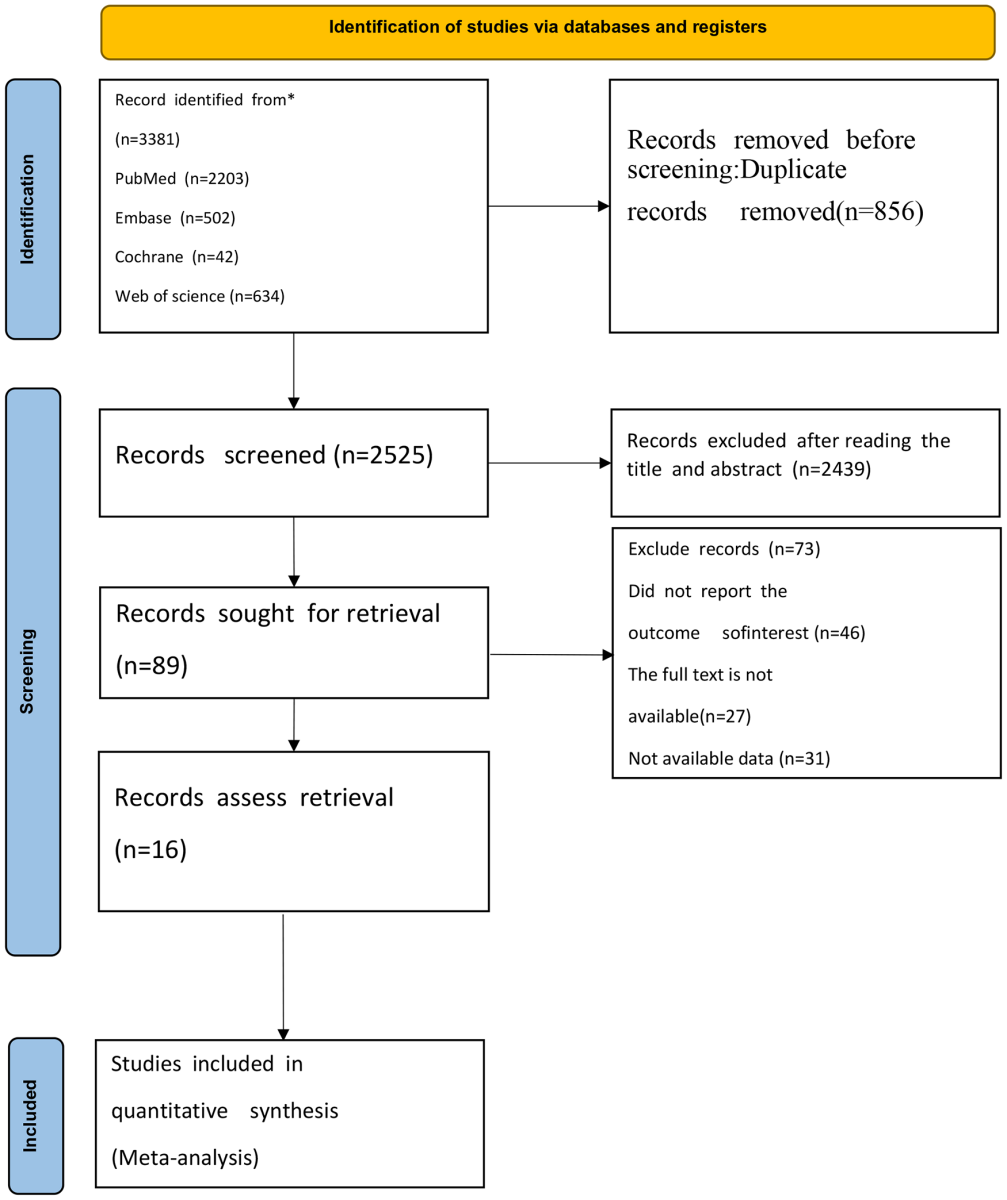
PRISMA 2020 flow diagram for new systematic reviews. n = number of records, PRISMA = preferred reporting items for systematic reviews and meta-analyses.

A systematic search of electronic databases was initially conducted, yielding a total of 3381 records. After removing 856 duplicate records, 2525 unique citations remained, which were then screened based on their titles and abstracts, leading to the exclusion of 2439 records that did not meet the predefined inclusion criteria. The full texts of the remaining 89 potentially eligible studies were retrieved and evaluated in detail, during which 73 studies were excluded. Ultimately, 16 studies met all the inclusion criteria and were included in the meta-analysis, with the detailed study selection process illustrated in Figure 1.

### 3.2. Study characteristics and quality evaluation results

The included 16 studies were published between 2010 and 2025 and involved a total of 31,543 elderly ED patients (mean age: 77.25 years). Delirium was diagnosed using validated tools, most commonly the CAM or its variants (including brief CAM, CAM-ICU, and modified chart-based CAM), with additional tools such as the abbreviated mental test 4, delirium triage screen, delirium observation screening scale, and diagnostic and statistical manual of mental disorders, 5th edition criteria also employed.

**Figure 2.**
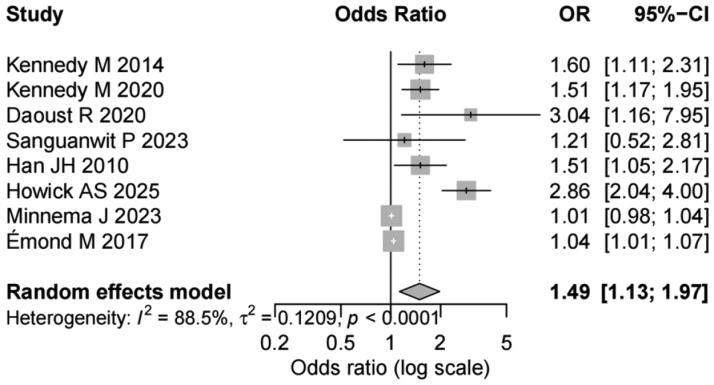
Forest plot of advanced age and delirium risk in elderly ED patients. CI = confidence interval, ED = emergency department, OR = odds ratio.

The majority of studies were conducted in North America and Europe, with only 1 study (Sanguanwit P 2023) performed in an Eastern region (Thailand). Delirium prevalence varied widely across studies, with relatively high rates reported in Turkey, the United States of America, and Thailand, and all included literature was of high quality (Table 1). (Fig. 2)

**Table 1:** Basic characteristics of the included studies.

Included study	Country	Study type	Core delirium assessment tool	Total sample size	Age	Delirium prevalence, no. (%)	Core risk factor categories	NOS
Kennedy M 2014^[11]^	USA	Prospective Observational Cohort Study	CAM	676	77	63 (9.3)	①②⑤	8
Han JH 2017^[12]^	USA	Prospective Cohort Study	Modified bCAM	228	74	105 (46.1)	①②	8
Kennedy M 2020^[13]^	USA	Multicenter Retrospective Cohort Study	CAM	817	77.7 ± 8.2	226 (28.0)	②	8
Béland E 2021^[14]^	Canada	Multicenter Prospective Cohort Study	CAM	612	78	68 (11.1)	②④	8
Daoust R 2020^[15]^	Canada	Multicenter Prospective Cohort Study	CAM	338	77 ± 8	41 (12.1)	②⑤	8
Cirbus J 2019^[16]^	USA	Prospective Cohort Study	bCAM, CAM-ICU	228	74	105 (46.1)	①②	8
Evensen S 2018^[17]^	Norway	Prospective Observational Study	DSM-5 Criteria	254	86.1 ± 5.2	49 (19.3)	②③	8
Fallon A 2018^[18]^	Ireland	Prospective Cohort Study	AMT4, CAM-ICU	198	78.8 ± 6.2	17 (8.6)	②	8
Han JH 2017^[19]^	USA	Prospective Cohort Study (Secondary Analysis)	CAM-ICU	1084	74.5	155 (14.3)	②	8
Émond M 2017^[20]^	Canada	Retrospective Cohort Study	Modified chart-based CAM	200	78.9 ± 7.3	36 (18.0)	②④	8
Han JH 2010^[21]^	USA	Prospective Cohort Study	CAM-ICU	628	76	108 (17.2)	②	8
Sanguanwit P 2023^[22]^	Thailand	Prospective Cohort Study	CAM-ICU	173	78 ± 8.1	49 (28.3)	②⑤	8
Howick AS 2025^[23]^	USA	Retrospective Cohort Study	DTS + bCAM	22,940	82.5	932 (4.1)	②⑤	8
Jordano JO 2023^[24]^	USA	Prospective Cohort Study (Secondary Analysis)	Modified bCAM	130	76	82 (63.1)	②⑥	8
Minnema J 2023^[25]^	Netherlands	Retrospective Cohort Study	DOSS + Clinical Evaluation	2794	79	602 (21.5)	②⑤	8
Boran Y 2024^[26]^	Turkey	Prospective Observational Study	bCAM	243	78	243 (100)	②	8

Risk factor category definitions: delirium etiological subtypes (e.g., metabolic disturbances, infections); baseline factors (e.g., female sex, dementia, functional dependence, cognitive decline, high comorbidity burden, medication history); environmental factors (e.g., overnight examinations); emergency department (ED)-related factors; clinical indicators (e.g., hypokalemia, high fall risk, ambulance transport); intervention-related factors (e.g., low-intensity PT/OT).

AMT4 = abbreviated mental test 4, bCAM = brief confusion assessment method, CAM = confusion assessment method, CAM-ICU = confusion assessment method for the intensive care unit, DOSS = delirium observation screening scale, DSM-5 = diagnostic and statistical manual of mental disorders, 5th edition, DTS = delirium triage screen, NOS = Newcastle–Ottawa scale, PT/OT = physical therapy/occupational therapy, USA = United States of America.

### 3.3. Risk factors for delirium

#### 3.3.1. Advanced age factors

**Figure 3.**
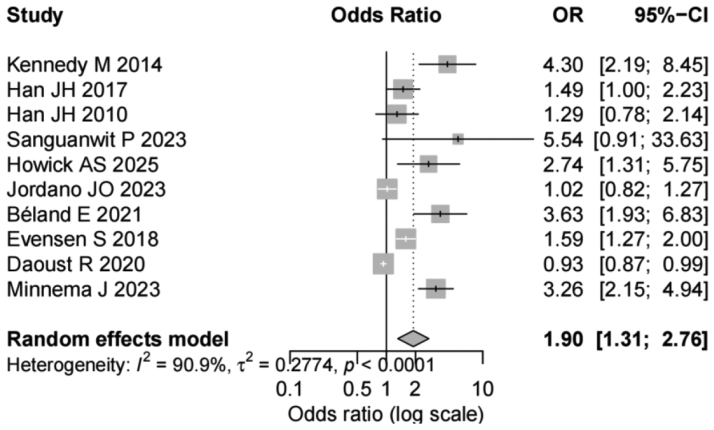
Forest plot of baseline cognitive impairment and delirium risk in elderly ED patients. CI = confidence interval, ED = emergency department, OR = odds ratio.

Advanced age was significantly associated with an increased risk of delirium in elderly patients presenting to the ED. After excluding studies reporting only standardized mean differences, a total of 8 observational studies were ultimately included in this meta-analysis to evaluate the association between advanced age and ED delirium risk. Using a random-effects model to account for substantial between-study heterogeneity, the pooled analysis demonstrated that advanced age was independently associated with a higher risk of delirium in this population (OR = 1.49, 95% CI: 1.13–1.97; *P* = .0049), with considerable statistical heterogeneity observed across the included studies (*I*^2^ = 88.5%). (Fig. 3)

#### 3.3.2. Baseline cognitive impairment factors

**Figure 4.**
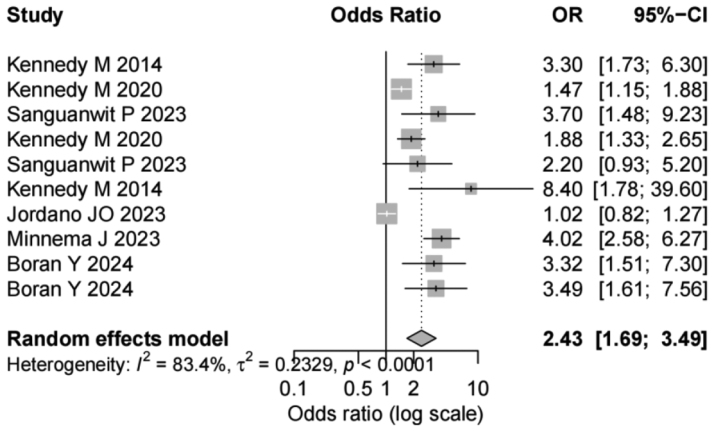
Forest plot of disease- or history-related and delirium risk in elderly ED patients. CI = confidence interval, ED = emergency department, OR = odds ratio.

Ten observational studies evaluating dementia, cognitive decline, or prior memory problems were included in the pooled analysis. Using a random-effects model, baseline cognitive impairment was significantly associated with an increased risk of delirium among elderly ED patients (OR = 1.90, 95% CI: 1.31–2.76). High heterogeneity was observed across studies (*I*^2^ = 90.9%). (Fig. 4)

#### 3.3.3. Disease- or history-related factors

**Figure 5.**
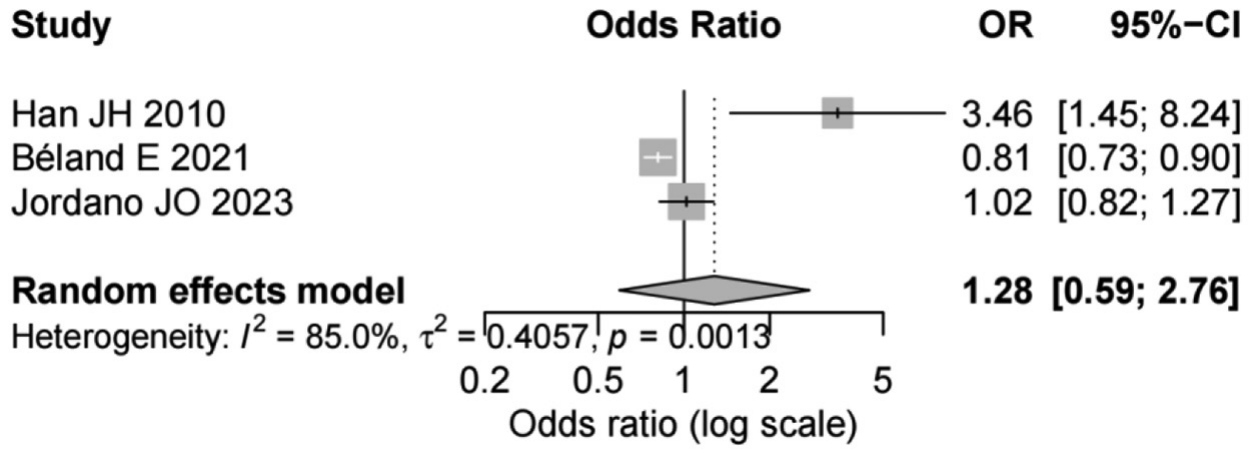
Forest plot of functional dependence and delirium risk in elderly ED patients. CI = confidence interval, ED = emergency department, OR = odds ratio.

Ten effect estimates from observational studies reporting disease- or history-related conditions (e.g., cerebrovascular disease, cardiovascular comorbidities, multimorbidity, and active malignancy) were included in the meta-analysis. Using a random-effects model, the presence of disease-related burden was significantly associated with an increased risk of delirium in elderly ED patients (pooled OR = 2.43, 95% CI: 1.69–3.49). Substantial heterogeneity was observed across studies (*I*^2^ = 83%, *P* < .001). (Fig. 5)

#### 3.3.4. Functional dependence and comorbidities factors

**Figure 6.**
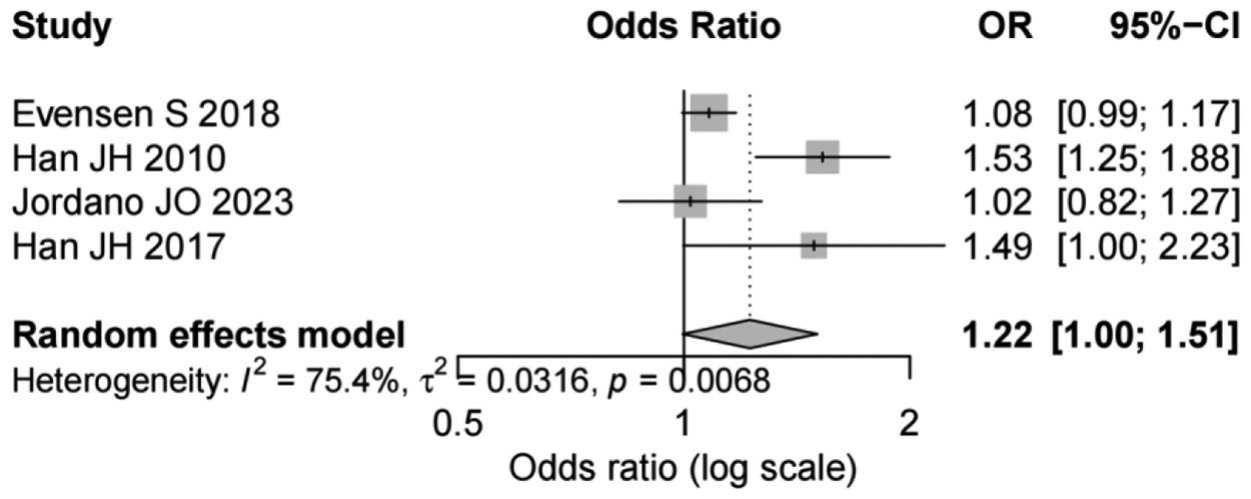
Forest plot of comorbidities and delirium risk in elderly ED patients. CI = confidence interval, ED = emergency department, OR = odds ratio.

Three studies evaluated functional dependence. The pooled random-effects analysis did not show a statistically significant association between functional status and delirium (pooled OR = 1.28, 95% CI: 0.59–2.76; *P* = .53), with substantial heterogeneity (*I*^2^ = 85.0%). Comorbidity burden was analyzed separately from disease-specific histories. Four studies examined comorbidity burden, and the pooled estimate suggested a borderline association with delirium (OR = 1.22, 95% CI: 1.00–1.51; *P* = .055), with considerable heterogeneity (*I*^2^ = 75.4%). (Fig. 6).

#### 3.3.5. ED-related factors and medication-related factors

**Figure 7.**
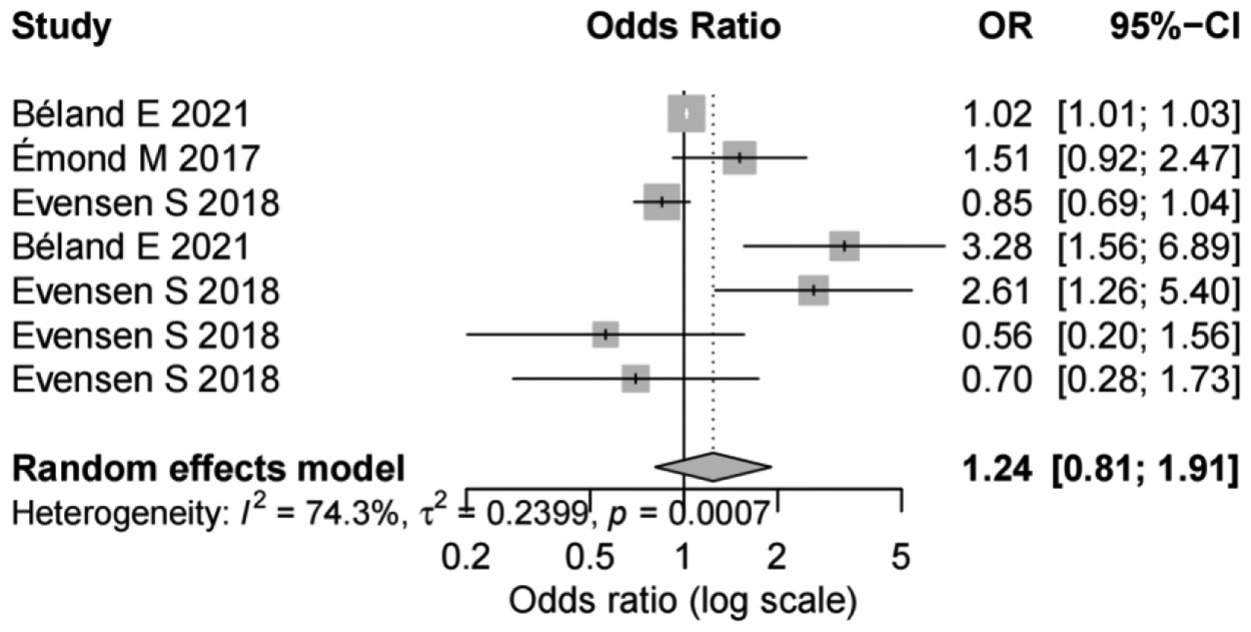
Forest plot of ED-related and delirium risk in elderly ED patients. CI = confidence interval, ED = emergency department, OR = odds ratio.

Seven studies investigated ED-related factors (e.g., crowding, boarding time, environmental or organizational features). The pooled analysis did not demonstrate a significant association with delirium (pooled OR = 1.24, 95% CI: 0.81–1.91; *P* = .32), although heterogeneity was substantial (*I*^2^ = 74.3%). Four studies examined potentially deliriogenic medication exposures. Medication-related factors were significantly associated with an increased risk of delirium (pooled OR = 1.49, 95% CI: 1.20–1.85; *P* = .0004), with no observed heterogeneity (*I*^2^ = 0.0%). (Fig. 7)

### 3.4. Publication bias for cognitive impairment

**Figure 8.**
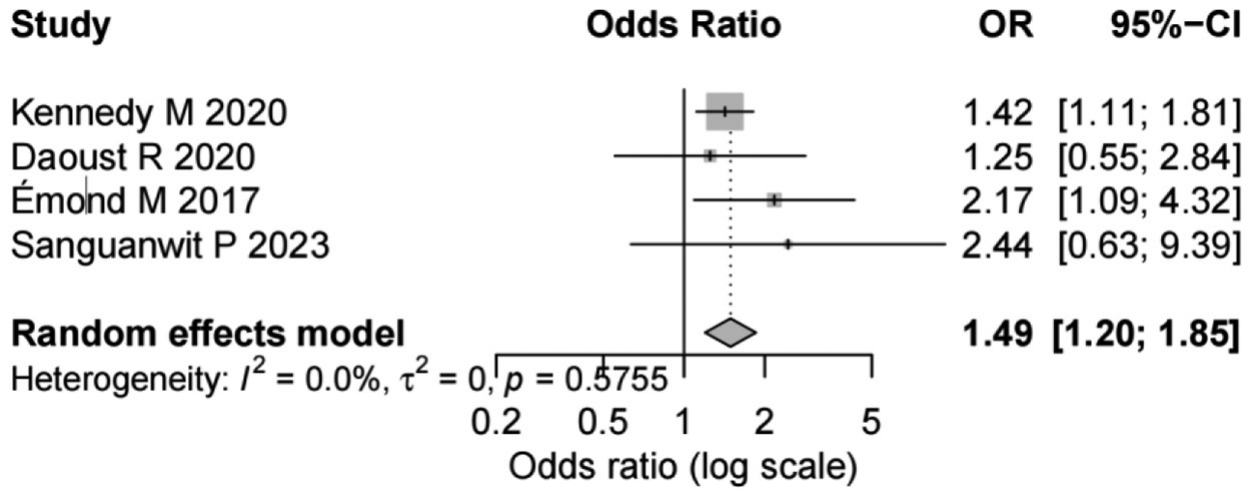
Forest plot of medication-related and delirium risk in elderly ED patients. CI = confidence interval, ED = emergency department, OR = odds ratio.

**Figure 9.**
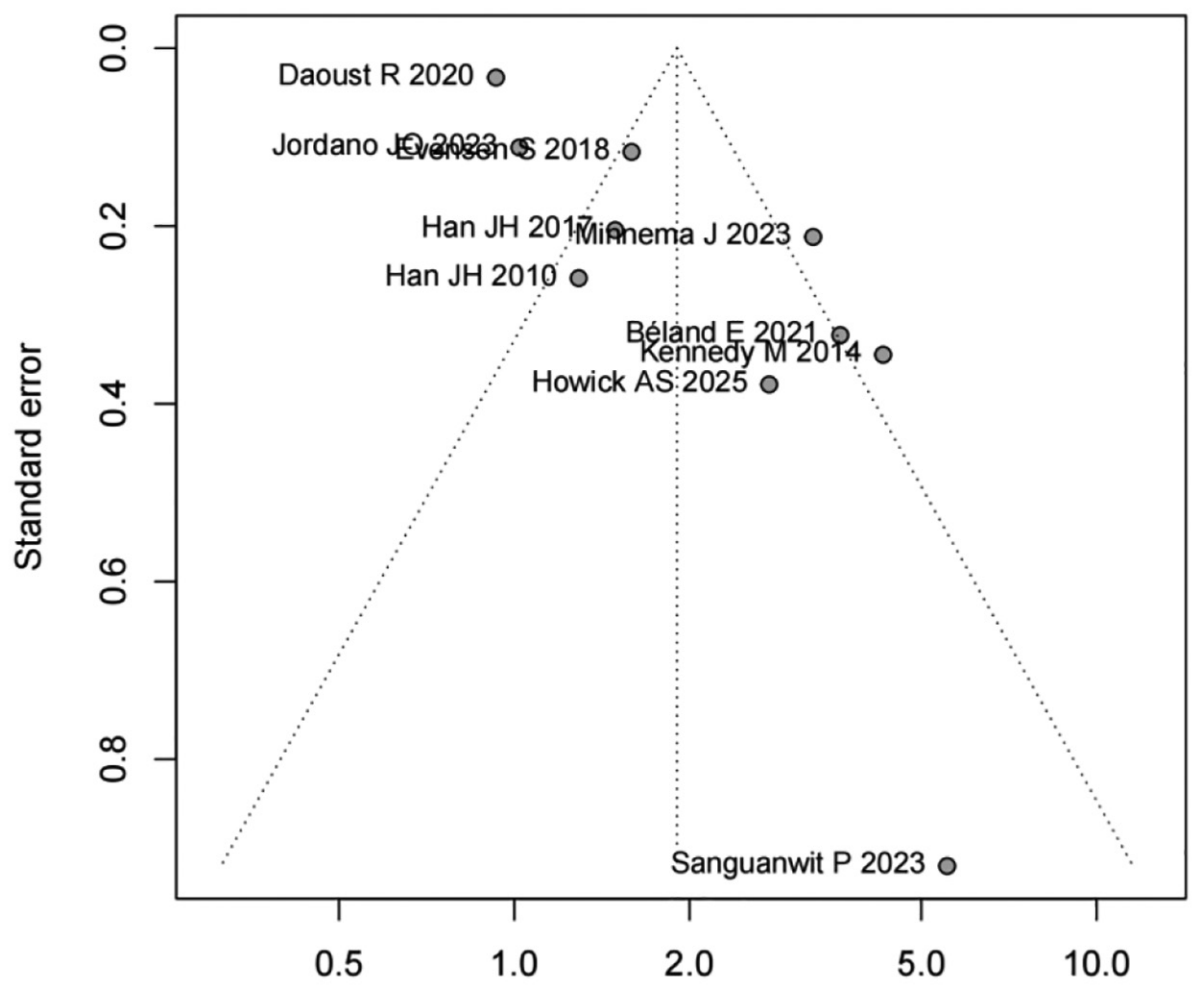
Cognitive impairment bias assessment.

For the domain of baseline cognitive impairment, visual inspection of the funnel plot did not reveal marked asymmetry, with most studies distributed symmetrically around the pooled effect size and within the pseudo 95% confidence limits. For baseline cognitive impairment, funnel plot inspection did not reveal marked asymmetry. Egger test did not indicate statistically significant publication bias. Because the number of studies was limited and heterogeneity was high, small-study effects cannot be completely excluded. (Figs. 8 and 9)

## 4. Discussion

In this systematic review and meta-analysis, we synthesized evidence on risk factors for delirium among elderly patients presenting to the ED. Across multiple domains, we found that baseline cognitive vulnerability and medical comorbidity were the strongest and most consistent predictors of ED delirium. Older age was also significantly associated with delirium, and patients with preexisting cognitive impairment (including dementia, prior memory problems, or documented cognitive decline) had almost a 2-fold higher risk of delirium. Disease- and history-related factors, such as prior cerebrovascular events and chronic neurological or cardiovascular disease, further increased the odds of delirium, whereas drug-related factors, particularly polypharmacy and psychoactive medication exposure, were associated with a more modest but significant elevation in risk. By contrast, pooled associations for global functional status, ED-environment variables, and infection/metabolic factors did not reach statistical significance, although effect estimates were directionally consistent with higher delirium risk.

These findings align closely with data from medically hospitalized older patients, in whom frailty, physical restraint, prior falls, severe illness, and cognitive impairment have been identified as key determinants of delirium, with ORs ranging from approximately 1.3 to 5.0.^[27]^ Together, these results support a vulnerability–precipitant model, in which age-related brain and systemic changes, reduced cognitive and functional reserve, and multimorbidity create a substrate on which acute ED stressors (such as infection, metabolic derangements, medication changes, or invasive procedures) precipitate delirium. The strong signal for cognitive impairment across both ED and inpatient settings also echoes mechanistic work in postoperative delirium, where preexisting neurodegeneration, chronic neuroinflammation, and reduced synaptic and vascular reserve have been proposed as central drivers of delirium susceptibility.^[28]^

The identification of partly modifiable risk factors (including polypharmacy, infection, severe illness markers, and sensory impairment) highlights concrete opportunities for intervention in the ED. Simple measures such as medication review to deprescribe high-risk agents, prompt evaluation and treatment of infection and metabolic disturbances, optimization of oxygenation and hemodynamics, and provision of visual and hearing aids may reduce delirium incidence in high-risk older adults. Environmental and process changes (minimizing tethers and restraints, reducing sensory overload, facilitating sleep, and encouraging family presence) are also supported by delirium prevention trials in inpatient settings and can be adapted to the ED context. Moreover, emerging perioperative literature points to the potential of digital cognitive screening platforms, frailty assessment tools, and machine learning–based risk scores to identify patients at particularly high risk; similar approaches could be translated into ED triage workflows to trigger early preventive bundles.

Several limitations should be considered. First, most included studies were observational, and adjusted models differed across studies. Second, heterogeneity was substantial in several domains, reflecting variation in patient populations, delirium assessment tools, timing and frequency of screening, exposure definitions, and analytic methods. Third, some risk-factor domains were informed by relatively few studies with wide CIs. Fourth, publication-bias tests have limited power when few studies are available. Finally, findings may not generalize to EDs with different patient populations, staffing models, or delirium screening practices.

### 4.1. Conclusion

Delirium among older ED patients is associated with advanced age, baseline cognitive impairment, and medication-related exposures. Comorbidity burden may also be associated with delirium, although the pooled estimate was borderline. These factors should be used as risk markers for early recognition and stratification rather than as evidence of causal drivers. Future prospective studies using standardized delirium assessment and consistent adjustment strategies are needed to clarify modifiable risk factors and develop ED-specific prevention pathways.

## Acknowledgments

The author wishes to express their gratitude to the team for their valuable contributions, help, and insightful discussions throughout the project.

## Author contributions

**Conceptualization:** Juan Mu, Liqun Zou.

**Data curation:** Juan Mu, Liqun Zou.

**Formal analysis:** Juan Mu, Liqun Zou.

**Funding acquisition:** Liqun Zou.

**Investigation:** Liqun Zou.

**Writing** – **original draft:** Liqun Zou.

**Writing** – **review & editing:** Liqun Zou.
